# Estimating Hospital Costs of Catheter-Associated Urinary Tract Infection

**DOI:** 10.1002/jhm.2079

**Published:** 2013-09-06

**Authors:** Edward H Kennedy, M Todd Greene, Sanjay Saint

**Affiliations:** 1 VA Center for Clinical Management Research, Ann Arbor VA Health Services Research and Development Center of ExcellenceAnn Arbor, Michigan; 2 Patient Safety Enhancement Program, Department of Internal Medicine, University of Michigan Health SystemAnn Arbor, Michigan; 3 Department of Biostatistics and Epidemiology, Center for Clinical Epidemiology and Biostatistics, University of PennsylvaniaPhiladelphia, Pennsylvania

## Abstract

Healthcare-associated infections are common, costly, and potentially deadly. However, effective prevention strategies are underutilized, particularly for catheter-associated urinary tract infection (CAUTI), one of the most common healthcare-associated infections. Further, since 2008, the Centers for Medicare and Medicaid Services no longer reimburses hospitals for the additional costs of caring for patients who develop CAUTI during hospitalization. Given the resulting payment pressures on hospitals stemming from this decision, it is important to factor in cost implications when attempting to encourage decision makers to support infection prevention measures. To this end, we present a simple tool (with easy-to-use online implementation) that hospitals can use to estimate hospital costs due to CAUTI, both before and after an intervention, to reduce inappropriate urinary catheterization. Using previously published cost and risk estimates, we show that an intervention yielding clinically feasible reductions in catheter use can lead to an estimated 50% reduction in CAUTI-related costs. Our tool is meant to complement the Society of Hospital Medicine's Choosing Wisely campaign, which highlights avoiding placement or continued use of nonindicated urinary catheters as a key area for improving decision making and quality of care while decreasing costs. *Journal of Hospital Medicine* 2013;8:519–522. © 2013 Society of Hospital Medicine

Healthcare-associated infections affect 5% to 10% of all hospitalized patients each year in the United States, account for nearly *45 billion in direct hospital costs, and cause nearly 100,000 deaths annually.^1^,^2^ Because catheter-associated urinary tract infection (CAUTI) is one of the most common healthcare-associated infections in the United States and is reasonably preventable, the Centers for Medicare and Medicaid Services stopped reimbursing hospitals in 2008 for the additional costs of caring for patients who develop CAUTI during hospitalization.^3^ Still, strategies for reducing inappropriate urinary catheterization are infrequently implemented in practice; this is despite a consensus that such strategies are effective. 4

To help motivate hospitals to reduce inappropriate urinary catheter use, we present a tool for estimating costs of CAUTI for individual hospitals. Although other tools for estimating the excess costs of healthcare-associated infections are available (eg, the APIC Cost of Healthcare-Associated Infections Model available at http://www.apic.org/Resources/Cost-calculators), they do not provide estimates of potential cost savings. Our approach adds to the literature by providing estimates of a hospital's current costs based on a few simple inputs (eg, annual admissions and catheterization rate), and also yields projected costs after a hypothetical intervention to prevent infections. Results are derived by combining appropriate cost and risk estimates from the literature. Importantly, an online implementation of our approach is available that can be easily used by clinicians, hospital administrators, and national policymakers. Our implementation nicely complements efforts like the Society of Hospital Medicine's Choosing Wisely campaign, which highlights avoiding inappropriate urinary catheter use first on its list of Five Things Physicians and Patients Should Question, and aims to increase awareness about issues that could improve patient outcomes and reduce healthcare costs.^5^ Although accounting for the full spectrum of institution-specific costs (eg, actual intervention costs, opportunity costs) was beyond the scope of this work, the simple tool we present helps meet the primary goal of generating an awareness of the potential cost savings stemming from CAUTI prevention efforts.

## Methods

### General Setup

**Figure 1:**
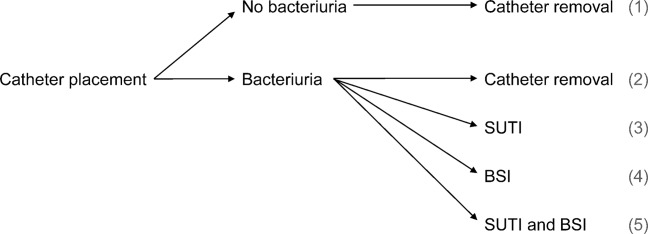
Possible patient trajectories. Abbreviations: BSI, bloodstream infection; SUTI, symptomatic urinary tract infection.

We consider 4 possible events after urinary catheter placement: bacteriuria, symptomatic urinary tract infection (SUTI), bloodstream infection (BSI), and catheter removal. Conservatively, assuming that bacteriuria must precede SUTI and BSI, there are 5 possible trajectories for any hospitalized patient (Figure 1): (1) no infection, (2) only bacteriuria, (3) bacteriuria and SUTI, (4) bacteriuria and BSI, or (5) bacteriuria, SUTI, and BSI. The cost of CAUTI for a particular hospital is therefore the per-patient cost of each trajectory multiplied by the number of patients experiencing each trajectory. Our approach for estimating hospital costs is based on factorizing the number of patients experiencing each trajectory into a product of terms for which estimates are available from the literature (see the Supporting Information, Appendix, in the online version of this article for all technical details).

### Deriving Estimates of Current Costs

We start with 2 minor simplifying assumptions. First, because the presence of asymptomatic bacteriuria is typically unknown, we only consider costs to the hospital due to SUTI and BSI 6; in other words, we assume hospitals do not incur costs for patients with trajectories 1 or 2. This assumption should only bias cost estimates conservatively. Second, we assume that patients with both SUTI and BSI (trajectory 5) incur costs equal to those for patients with only BSI (trajectory 4). Further, because the joint risk of SUTI and BSI is unknown, we conservatively assume SUTI must precede BSI. Under these assumptions we can write: (total CAUTI costs) = (per-patient SUTI cost) × (number with SUTI but no BSI) + (perpatient BSI cost) × (number with BSI).

We use per-patient hospital costs of SUTI and BSI of $911 and $3824, respectively, which were determined using a microcosting approach 6 and adjusted for inflation using the general Consumer Price Index.^7^ Although an alternative strategy for estimating costs would be to enter the hospital-specific, per-patient costs of SUTI and BSI into the above equation, these quantities are often difficult to measure or otherwise unavailable. Thus, it remains to factorize the number of hospitalized patients who develop SUTI and BSI into component terms for which we have accessible estimates. First note that the number with only SUTI (or any BSI) equals the total number of patients hospitalized times the proportion of hospitalizations with only SUTI (or any BSI). The former quantity depends on the particular hospital and so is specified as an input by the user. The latter quantity can be factorized further under our aforementioned conservative assumption that bacteriuria must precede SUTI and BSI.

Specifically, for SUTI: (Proportion SUTI but no BSI) = {(SUTI risk among those catheterized with bacteriuria) − (BSI risk among those catheterized with bacteriuria)} × (bacteriuria risk among those catheterized) × (proportion catheterized).

And for BSI: (Proportion BSI) = (BSI risk among those catheterized with bacteriuria) × (bacteriuria risk among those catheterized) × (proportion catheterized).

The risks of SUTI and BSI among those catheterized with bacteriuria, along with the risk of bacteriuria among those catheterized, have been estimated previously via a meta-analytic approach.2000 The proportion catheterized depends on the particular hospital, such as the total number of patients hospitalized, and so is also specified as a user input. Therefore, we have now factorized the total hospital costs due to CAUTI as a product of either user-specified terms or terms for which we have estimates from the literature. All estimates and corresponding standard errors derived from the literature are listed together in Table 1 (see the Supporting Information, Appendix Section 1, for further details in the online version of this article).

**Table 1:** Input Values Used in Estimating Hospital Costs Due to Catheter-Associated Urinary Tract Infection

Quantity	Estimate (SE)
Overall risk of bacteriuria among those catheterized	26.0% (1.53%)
Per-day risk of bacteriuria among those catheterized	5.0%
days	6.68
Risk of SUTI among those catheterized with bacteriuria	24.0% (4.08%)
Risk of BSI among those catheterized with bacteriuria	3.6% (0.10%)
Per-patient SUTI cost	$911 ($911)
Per-patient BSI cost	$3824 ($3824)

NOTE: Abbreviations: BSI, bloodstream infection; SE, standard error; SUTI, symptomatic urinary tract infection. Most values were derived from or originally published in Saint (2000).6 Costs were inflation adjusted using the general Consumer Price Index.

### Deriving Projected Costs After Intervention

The approach described above permits estimation of current costs for managing patients with CAUTI for a particular hospital. To estimate projected costs after participation in an intervention to reduce infection risk, we characterize interventions of interest and introduce additional factorization. Specifically, following previous work,^8^ we consider interventions that reduce (1) placement (ie, the proportion catheterized) and (2) duration (ie, the mean duration of catheterization). Incorporating reductions in placement is straightforward, because our above expression for costs already contains a term for the proportion catheterized. However, incorporating reductions in duration requires further factorization. Under the assumptions of constant per-day risks of bacteriuria and of catheter removal, we can write the postintervention risk of bacteriuria among the catheterized as a function of (1) the percent decrease in mean duration of catheterization due to intervention, and (2) the preintervention risk of bacteriuria among the catheterized (see the Supporting Information, Appendix Section 2, for further details in the online version of this article). This means we can fully characterize postintervention costs as a function of user-specified quantities, quantities specific to the intervention (which are varied across plausible ranges), and quantities for which we have estimates from the literature. Therefore, we can estimate savings by subtracting postintervention costs from current costs.

Because our estimators of current costs, projected costs, and savings are all formulated as functions of other estimators, we use the standard delta method approach 9 to derive appropriate variance estimates (see the Supporting Information, Appendix Section 3, for further details in the online version of this article).

### Online Implementation

Customized results (based on annual admissions, urinary catheter prevalence, and other inputs) can be computed using online implementation of our proposed method at http://cauti.umms.med.umich.edu/PHP/CAUTI_input.php. Although the work presented in this article incorporates risk and cost estimates from the literature whenever possible, the online implementation allows full user specification of input values.

## RESULTS

**Figure 2:**
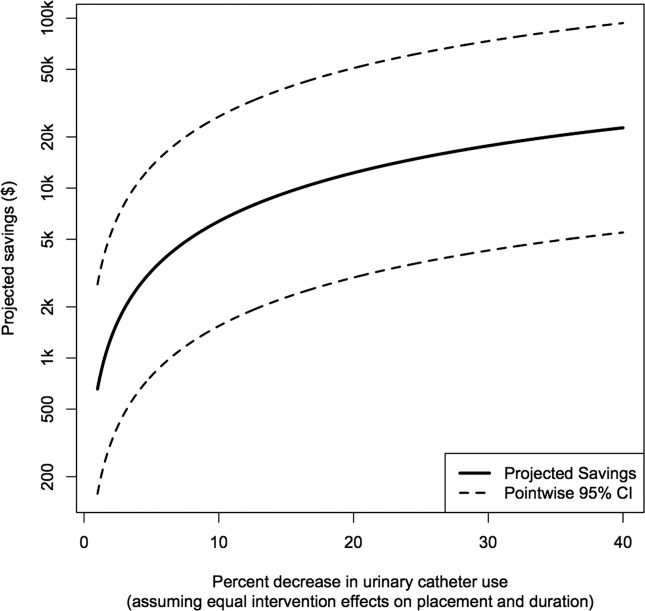
Projected cost savings for a hypothetical hospital projections were generated for a hypothetical hospital with 3000 total patients and 15% having urinary catheters (ie, 450 patients catheterized prior to intervention) and with default values given in Table 1. Abbreviations: CI, confidence interval.

Figure 2 shows the projected savings in hospital costs due to CAUTI across a range of interventions defined by percent decreases in placement and duration, for a hypothetical hospital with 3000 total patients, 15% with urinary catheters preintervention, and with all other default values listed in Table 1. The current costs for this hospital (ie, the costs when the percent reduction in placement and duration is zero) are estimated to be $37,868 (95% confidence interval [CI]: $9159-$156,564). After an intervention resulting in 40% reductions in both urinary catheter placement and duration, this hospital would be expected to save $22,653 (95% CI: $5479-$93,656). A less effective intervention yielding a 10% reduction in both urinary catheter placement and duration would result in more modest savings of $6376 (95% CI: $1542-$26,360).

After an intervention resulting in 29% and 37% reductions in placement and duration, respectively, reflecting reductions seen in practice,^10^,^11^ our hypothetical hospital is estimated to save $19,126 (95% CI: $4626-$79,074). This reflects an estimated savings of nearly 50%.

## DISCUSSION

We have presented a tool for estimating customized hospital costs of CAUTI, both before and after a hypothetical intervention to reduce risk of infection. Our approach relies on mostly conservative assumptions, incorporates published risk estimates (properly accounting for their associated variability), and has easy-to-use online implementation. We believe this can play an important role in motivating hospitals to reduce inappropriate urinary catheter use.

The methodology employed here does have a few limitations. First and foremost, our results depend on the reliability of the input values, which are either provided by users or are based on estimates from the literature (see Table 1 for a complete list of suggested defaults). New information could potentially be incorporated if and when available. For example, substitution of more precise risk estimates could help reduce confidence interval length. Second, our approach essentially averages over hospital quality; we do not directly take into account quality of care or variation in underlying infection risk across hospitals in computing estimated costs. Finally, we only compute direct costs due to infection; other costs (eg, intervention costs) would typically also need to be considered for decision making.

Despite these limitations, we believe that our tool can help infection control professionals demonstrate the values of CAUTI prevention efforts to key administrators, particularly at a time where it has become increasingly necessary to develop a business case to initiate new interventions or justify the continued support for ongoing programs.^12^ Additionally, we believe the proposed approach can be an important supplement to initiatives like the Society of Hospital Medicine's Choosing Wisely campaign, which aims to help reduce inappropriate urinary catheter use. Reducing catheter utilization has the potential to reduce costs associated with caring for CAUTI patients, but more importantly would help reduce CAUTI incidence as well as catheter-related, noninfectious complications.^13^,^14^ We hope that our tool will greatly assist hospitals in promoting their CAUTI prevention efforts and improve the overall safety of hospitalized patients.

## Notes

Disclosures: This project was supported by the Ann Arbor VA Medical Center/University of Michigan Patient Safety Enhancement Program (PSEP) and a subcontract to implement multistate CAUTI prevention with the Agency for Healthcare Research and Quality/Health Educational and Research Trust. Mr. Kennedy has no conflicts of interest to report. Drs. Saint and Greene are subcontracted to implement multistate CAUTI prevention with the Agency for Healthcare Research and Quality/Health Educational and Research Trust. Dr. Saint has received numerous honoraria and speaking fees for lectures on healthcare-associated infection prevention, implementation science, and patient safety from hospitals, academic medical centers, professional societies, and nonprofit foundations. None of these activities are related to speaker's bureaus. Dr. Saint is also on the medical advisory board of Doximity, a new social networking site for physicians. The views expressed in this article are those of the authors and do not necessarily reflect the position or policy of the Department of Veterans Affairs.
