# Supplementary material for: Estimating Hospital Costs of Catheter-Associated Urinary Tract Infection
Source: J Hosp Med. 2013 Sep 6;8(9):519–22. doi: 10.1002/jhm.2079 (PMC3786530; doi:10.1002/jhm.2079)
Supplement: Supplementary file 1 [file jhm0008-0519-SD1.doc]

**Journal of Hospital Medicine Author Contribution Form**

The *Journal of Hospital Medicine* adheres to the authorship guidelines set by the International Committee of Medical Journal Editors – “Authorship should be based on: (1) substantial contributions to conception and design, acquisition of data, or analysis and interpretation of data; (2) drafting the article or revising it critically for important intellectual content; and (3) final approval of the version to be published. Authors should meet conditions 1, 2, and 3” (from [www.icmje.org](http://www.icmje.org/)). Authorship is not justified for individuals who simply obtain or provide funding, participate in data collection or general supervision of the research, or serve as head of the group, though such contributors may be acknowledged with their written permission in the Acknowledgements section.

Completion of the author contribution form is not required upon initial manuscript submission. If a revision is requested after initial peer review, the corresponding author should complete this authorship form on behalf of all authors and upload it as Supplementary Material Not for Review. It will appear online as Supporting Information if the article is published. Failure to upload this form will block further manuscript processing.

Corresponding Author: Sanjay Saint Manuscript Number: JHM-13-0147

*(Please print)*

Manuscript Title: ESTIMATING HOSPITAL COSTS OF CATHETER-ASSOCIATED URINARY TRACT INFECTION

Please list authors in the order they appear in the submission.

Author Name: Edward Kennedy

| 1**. (Check at least 1 below):** | 2. **(Check at least 1 below):** | 3. **(Check below to affirm approval):** |
| --- | --- | --- |
| X Conception and design | X Drafting of the manuscript | X Final approval of the manuscript |
| □ Acquisition of data | X Critical revision of the manuscript   for important intellectual content |  |
| X Analysis and interpretation of data |  |  |

Author Name: M. Todd Greene

| 1**. (Check at least 1 below):** | 2. **(Check at least 1 below):** | 3. **(Check below to affirm approval):** |
| --- | --- | --- |
| X Conception and design | X Drafting of the manuscript | X Final approval of the manuscript |
| □ Acquisition of data | X Critical revision of the manuscript   for important intellectual content |  |
| X Analysis and interpretation of data |  |  |

Author Name: Sanjay Saint

| 1**. (Check at least 1 below):** | 2. **(Check at least 1 below):** | 3. **(Check below to affirm approval):** |
| --- | --- | --- |
| X Conception and design | □ Drafting of the manuscript | X Final approval of the manuscript |
| □ Acquisition of data | X Critical revision of the manuscript   for important intellectual content |  |
| □ Analysis and interpretation of data |  |  |
