# Supplementary material for: Estimating Hospital Costs of Catheter-Associated Urinary Tract Infection
Source: J Hosp Med. 2013 Sep 6;8(9):519–22. doi: 10.1002/jhm.2079 (PMC3786530; doi:10.1002/jhm.2079)
Supplement: Supplementary file 2 [file jhm0008-0519-SD2.pdf]

1 Notation

Define the following, with $a = 0$ indicating pre-intervention quantities and $a = 1$ indicating post-intervention quantities:

$$\pi^a_{u|c} = \pi_{u|c}(m^a_c, r_u) = \text{overall risk of bacteriuria among those catheterized}$$
$$m^a_c = \text{mean duration of catheterization}$$
$$r_u = \text{per-day risk of bacteriuria among those catheterized}$$
$$\pi_{s|c,u} = \text{risk of SUTI among those catheterized with bacteriuria}$$
$$\pi_{b|c,u} = \text{risk of BSI among those catheterized with bacteriuria}$$
$$n = \text{total number of patients hospitalized}$$
$$p^a_c = \text{proportion of patients catheterized}$$
$$c_s = \text{per-patient SUTI costs}$$
$$c_b = \text{per-patient BSI costs}$$

Let C^0 and C^1 be the total pre- and post-intervention hospital costs due to CAUTI, respectively; these are functions of the above quantities. Our goal is to derive estimates and confidence intervals for the expected savings after intervention, defined as $S = C^0 - C^1$.

From the literature we have estimates and standard errors for the parameters $\pi^0_{u|c}$, $\pi_{s|c,u}$, and $\pi_{b|c,u}$, along with point estimates of r_u, c_s, and c_b (we assume the means and standard errors for the costs are equal, although this assumption can be varied in sensitivity analyses):

$$\hat{\pi}^0_{u|c} = 26.0\%,\ \widehat{se}(\hat{\pi}^0_{u|c}) = 1.53\%$$
$$r_u = 5.0\%$$
$$\hat{\pi}_{s|c,u} = 24.0\%,\ \widehat{se}(\hat{\pi}_{s|c,u}) = 4.08\%$$
$$\hat{\pi}_{b|c,u} = 3.6\%,\ \widehat{se}(\hat{\pi}_{b|c,u}) = 0.10\%$$
$$\hat{c}_s = \widehat{se}(\hat{c}_s) = \$911$$
$$\hat{c}_b = \widehat{se}(\hat{c}_b) = \$3824$$

The quantities n and p^0_c require user-specified values. We characterize interventions by their effects on m^0_c (duration) and p^0_c (placement), and thus explore results across ranges for m^1_c and p^1_c.

We make a number of assumptions in the interest of deriving conservative cost estimates. First, we assume that catheterization is a necessary condition for bacteriuria, and that bacteriuria is a necessary condition for both SUTI and BSI. We also assume costs for patients with SUTI and BSI are the same as those for patients with only BSI. And since the joint risk of SUTI and BSI is not well understood, we further assume that SUTI is a necessary condition for BSI. Under these assumptions, the pre- and post-intervention costs are:

$$C^a = n\Big\{c_s(\pi_{s|c,u} - \pi_{b|c,u})\pi^a_{u|c}p^a_c + c_b\pi_{b|c,u}\pi^a_{u|c}p^a_c\Big\} = n\Big\{c_s(\pi_{s|c,u} - \pi_{b|c,u}) + c_b\pi_{b|c,u}\Big\}p^a_c\pi^a_{u|c}$$

Therefore the savings can be expressed as:

$$S = C^0 - C^1 = n\Big\{c_s(\pi_{s|c,u} - \pi_{b|c,u}) + c_b\pi_{b|c,u}\Big\}\Big(p_c^0\pi_{u|c}^0 - p_c^1\pi_{u|c}^1\Big)$$

2 Bacteriuria and catheterization duration

The risk of bacteriuria among those catheterized is frequently characterized in the literature as a per-day risk (typically $r_u = 5.0\%$). Under this assumption, along with the assumption that catheterization is a necessary condition for bacteriuria and that among those catheterized there is a $(1/m_c)$ chance per day of having the catheter removed, it follows that:

$$\pi_{u|c} = \sum_{d=1}^{\infty}\left\{1-(1-r_u)^d\right\}\left(1-\frac{1}{m_c}\right)^{d-1}\left(\frac{1}{m_c}\right) = \frac{r_u m_c}{1+r_u(m_c-1)}$$

since $\{1-(1-r_u)^d\}$ is the risk of bacteriuria given a catheterization duration of d days and since $\{1-(1/m_c)\}^{d-1}(1/m_c)$ is the chance of being catheterized d days.

If we characterize the post-intervention duration of catheterization m_c^1 in terms of a percent decrease k_d relative to the pre-intervention duration so that $m_c^1 = (1-k_d)m_c^0$, then:

$$\pi_{u|c}^1 = \frac{r_u m_c^1}{1+r_u(m_c^1-1)} = \frac{r_u(1-k_d)m_c^0}{1+r_u\{(1-k_d)m_c^0-1\}} = \frac{(1-k_d)\pi_{u|c}^0}{1-k_d\pi_{u|c}^0}$$

3 Estimation and inference

As with post-intervention duration of catheterization, we can characterize the post-intervention proportion catheterized p_c^1 in terms of a percent decrease k_p relative to the pre-intervention proportion catheterized, so that $p_c^1 = (1-k_p)p_c^0$. Then based on the above work we can write the savings as:

$$S = C^0 - C^1 = n\Big\{c_s(\pi_{s|c,u} - \pi_{b|c,u}) + c_b\pi_{b|c,u}\Big\}\Big\{p_c^0\pi_{u|c}^0 - (1-k_p)p_c^0\frac{(1-k_d)\pi_{u|c}^0}{1-k_d\pi_{u|c}^0}\Big\}$$

Therefore an estimate for the savings is given by:

$$\hat{S} = n\Big\{\hat{c}_s(\hat{\pi}_{s|c,u} - \hat{\pi}_{b|c,u}) + \hat{c}_b\hat{\pi}_{b|c,u}\Big\}\Big\{p_c^0\hat{\pi}_{u|c}^0 - \frac{(1-k_p)(1-k_d)p_c^0\hat{\pi}_{u|c}^0}{1-k_d\hat{\pi}_{u|c}^0}\Big\}$$

We now have an estimator for S that depends only on either fixed known quantities (e.g., n and p_c^0, which are user-specified, and k_p and k_d, which are varied across ranges of possible values) or on quantities for which we have estimates and variances from the literature (these can also be varied by the user). Thus point estimates for S are immediately available.

For variance estimates we can use the delta method. In order to ensure that confidence intervals respect the fact that S is greater than zero, we derive variances on the log scale.

First note that for $g(\pi^0_{u|c}) = p^0_c\hat{\pi}^0_{u|c} - \frac{(1-k_p)(1-k_d)p^0_c\hat{\pi}^0_{u|c}}{1-k_d\hat{\pi}^0_{u|c}}$ we have $g'(\pi^0_{u|c}) = p^0_c\{1 - \frac{(1-k_p)(1-k_d)}{(1-k_d\hat{\pi}^0_{u|c})^2}\}$.

Therefore, by the delta method:

$$\widehat{\text{Var}}\Big\{p^0_c\hat{\pi}^0_{u|c} - \frac{(1-k_p)(1-k_d)p^0_c\hat{\pi}^0_{u|c}}{1-k_d\hat{\pi}^0_{u|c}}\Big\} \approx \left[p^0_c\Big\{1 - \frac{(1-k_p)(1-k_d)}{(1-k_d\hat{\pi}^0_{u|c})^2}\Big\}\right]^2 \widehat{\text{Var}}(\hat{\pi}^0_{u|c})$$

Let $\pi^*_{s|c,u} = \pi_{s|c,u} - \pi_{b|c,u}$. Then, for the function:

$$h\Big(\pi_{s|c,u}, \pi_{b|c,u}, g(\pi^0_{u|c})\Big) = \log S = \log n + \log(c_s\pi^*_{s|c,u} + c_b\pi_{b|c,u}) + \log g(\pi^0_{u|c})$$

the gradient $\nabla h\Big(c_s, c_b, \pi_{s|c,u}, \pi_{b|c,u}, g(\pi^0_{u|c})\Big)$ is:

$$\Big(c_s\pi^*_{s|c,u} + c_b\pi_{b|c,u}\Big)^{-1}\Big\{\pi^*_{s|c,u},\ \pi_{b|c,u},\ c_s,\ (c_b - c_s),\ \frac{g'(\pi^0_{u|c})}{g(\pi^0_{u|c})}(c_s\pi^*_{s|c,u} + c_b\pi_{b|c,u})\Big\}$$

Let $c^*_b = c_b - c_s$, with $\pi^*_{b|c,u} = \pi_{b|c,u}$ and $c^*_s = c_s$ for notational convenience. Then, assuming the estimates $\hat{c}_s$, $\hat{c}_b$, $\hat{\pi}_{s|c,u}$, $\hat{\pi}_{b|c,u}$, and $g(\hat{\pi}^0_{u|c})$ are all uncorrelated, another application of the delta method implies:

$$\widehat{\text{Var}}(\log \hat{S}) \approx \frac{\sum_{j\in\{s,b\}}\Big\{\hat{\pi}^{*2}_{j|c,u}\widehat{\text{Var}}(\hat{c}_j) + \hat{c}^{*2}_j\widehat{\text{Var}}(\hat{\pi}_{j|c,u})\Big\}}{(\hat{c}_s\hat{\pi}^*_{s|c,u} + \hat{c}_b\hat{\pi}_{b|c,u})^2} + \left\{\frac{g'(\hat{\pi}^0_{u|c})^2}{g(\hat{\pi}^0_{u|c})}\right\}^2 \widehat{\text{Var}}(\hat{\pi}^0_{u|c})$$

And a 95% confidence interval for S is given by:

$$\exp\left[\log\hat{S} \pm 1.96\sqrt{\frac{\sum_{j\in\{s,b\}}\Big\{\hat{\pi}^{*2}_{j|c,u}\widehat{\text{Var}}(\hat{c}_j) + \hat{c}^{*2}_j\widehat{\text{Var}}(\hat{\pi}_{j|c,u})\Big\}}{(\hat{c}_s\hat{\pi}^*_{s|c,u} + \hat{c}_b\hat{\pi}_{b|c,u})^2} + \left\{\frac{g'(\hat{\pi}^0_{u|c})^2}{g(\hat{\pi}^0_{u|c})}\right\}^2 \widehat{\text{Var}}(\hat{\pi}^0_{u|c})}\right]$$
